# Gene flow and population structure in the Mexican blind cavefish complex (*Astyanax mexicanus*)

**DOI:** 10.1186/1471-2148-12-9

**Published:** 2012-01-23

**Authors:** Martina Bradic, Peter Beerli, Francisco J García-de León, Sarai Esquivel-Bobadilla, Richard L Borowsky

**Affiliations:** 1 Instituto de Tecnologia Química e Biológica, Universidade Nova de Lisboa, (Av. da República), Oeiras, (2780-157), Portugal; 2 Biology Department, New York University, (100 Washington Square East), NYC, 10003, USA; 3 Department of Scientific Computing, Florida State University, (150-T Dirac Science Library), Tallahassee, (32306-4120), USA; 4 Laboratorio de Genética para la Conservación, Centro de Investigaciones Biologicas del Noroeste La Paz, (Mar Bermejo #195), La Paz, (CP. 23090), Mexico

## Abstract

**Background:**

Cave animals converge evolutionarily on a suite of troglomorphic traits, the best known of which are eyelessness and depigmentation. We studied 11 cave and 10 surface populations of *Astyanax mexicanus *in order to better understand the evolutionary origins of the cave forms, the basic genetic structuring of both cave and surface populations, and the degree to which present day migration among them affects their genetic divergence.

**Results:**

To assess the genetic structure within populations and the relationships among them we genotyped individuals at 26 microsatellite loci. We found that surface populations are similar to one another, despite their relatively large geographic separation, whereas the cave populations are better differentiated. The cave populations we studied span the full range of the cave forms in three separate geographic regions and have at least five separate evolutionary origins. Cave populations had lower genetic diversity than surface populations, correlated with their smaller effective population sizes, probably the result of food and space limitations. Some of the cave populations receive migrants from the surface and exchange migrants with one another, especially when geographically close. This admixture results in significant heterozygote deficiencies at numerous loci due to Wahlund effects. Cave populations receiving migrants from the surface contain small numbers of individuals that are intermediate in both phenotype and genotype, affirming at least limited gene flow from the surface.

**Conclusions:**

Cave populations of this species are derived from two different surface stocks denoted "old" and "new." The old stock colonized caves at least three times independently while the new stock colonized caves at least twice independently. Thus, the similar cave phenotypes found in these caves are the result of repeated convergences. These phenotypic convergences have occurred in spite of gene flow from surface populations suggesting either strong natural or sexual selection for alleles responsible for the cave phenotype in the cave environment.

## Background

The mechanisms underlying the evolution of convergent phenotypes in independent natural populations pose long-standing questions in evolutionary biology. The extent to which convergent or parallel changes draw on preexisting genetic variation in ancestral populations versus new mutations is still debated [1,2]. The molecular and genetic changes that underly most convergences are still unknown.

Convergence is also of interest to evolutionists because it provides an element of replication to evolutionary studies that is often otherwise absent. Replication allows for the powerful testing of evolutionary hypotheses. Cave-dwelling organisms provide the best known examples of convergences, sharing similar phenotypes such as loss of eyes and pigmentation across diverse taxonomic groups [3-5].

The Mexican blind cavefish (*Astyanax mexicanus*) is nearly unique among cave animals because the cave forms have closely related surface conspecifics and the two forms are fully interfertile [6]. The ability to hybridize the cave and surface forms permits the genetic analysis of the factors involved in cave adaptation. There are 29 known cave populations of this species dispersed over three geographically distinct areas, thus this group may contain multiple examples of convergence [7].

Each population inhabits a food and light restricted cave environment. Members of these populations exhibit numerous cave-related evolutionary traits, including reduction in pigment and eye size, hypertrophy of non-optic sensory organs, increased condition factor, and robust patterns of reduced sleep; presumably all are evolved in response to perpetual darkness and reduced food availability [6-10].

Thus, the cave colonizations of *Astyanax *populations provide replicates of an excellent "natural experiment" which allows us to address important evolutionary questions, including the extent to which evolutionary changes in morphology, behavior and physiology are driven by selection versus drift [11-13]. These two alternatives can be distinguished in a number of ways in this system, but any determination will require an understanding of the underlying demography of the populations as well as a clarification of the relationships among them.

**Figure 1:**
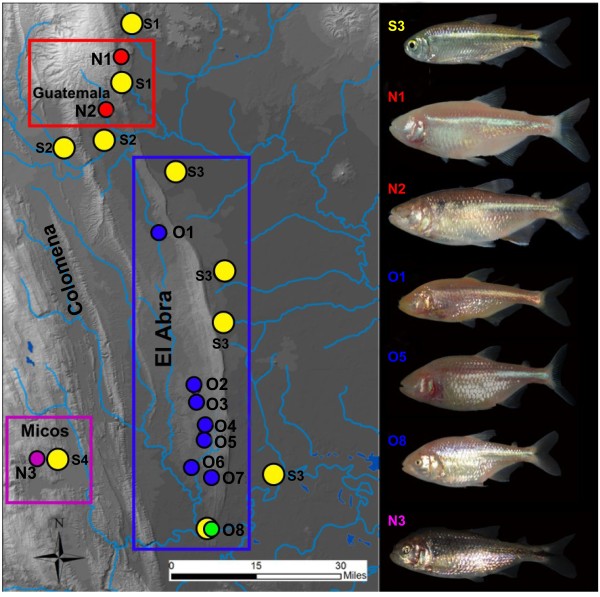
**Map of the Sierra de El Abra region showing all the cave and surface collection sites**. Colored boxes delineate major geographical regions (labeled below), as following: El Abra region: O1 - O8 (blue & green circles); Guatemala region: N1 - N2 (red circles); Micos region: N3 (purple circles); Surface localities S1 - S4 (yellow circles). Light blue lines represent different river systems in the area.

Previous phylogeographic studies of *Astyanax *cavefish, using microsatellite and mtDNA, showed that the cave populations are derived from at least two different surface stocks that inhabited the Sierra de El Abra and nearby regions in succession [14-17]. The estimates from mtDNA suggest that these two groups diverged about 6.7 Mya [15]. Surface forms of the older stock originally inhabited the rivers in the El Abra region and were the likely ancestors of a series of cave populations, which we designate as "old." Subsequently, the surface fish of the old stock went locally extinct. The region was then invaded by another stock of *A. mexicanus*, which gave rise to the current surface populations and a second set of cave populations we designate as "new" (Figure 1) [14,16,17].

While previous studies revealed that the extant cave populations were derived from a minimum of two ancestral stocks, there may have been more. In addition, the question of how many independent invasions of the underground led to the present day *Astyanax *cave fauna remains unanswered. To understand the demographic component of the phenotypic evolution we studied cave populations from the full extent of their known distribution. We give a detailed description of genetic differentiation in multiple cave populations and their relatedness with surface morphs, and estimate effective population sizes and the rates of gene flow among select populations based on multiple independent markers.

## Methods

### Sampling

All fish specimens were collected in March 2008 and preserved in 70% ethanol. A total of 568 *Astyanax *samples were taken from 11 cave and 10 surface locations. The cave populations sampled can be divided into three geographically distinct regions, which span the full geographic range of the cave forms. The first is the Sierra de El Abra cave cluster, which is the most extensive of the regions. To its west is the second region, Micos, on the Western slope of the Sierra de Colmena and finally, to the north of the El Abra, is the Sierra de Guatemala region.

The El Abra cave cluster is represented in our study by eight caves (from North to South, O1 to O8): Pachón, Yerbaniz, Japones, Arroyo, Tinaja, Curva, Toro, and Chica, respectively. In the Sierra de Guatemala we sampled two caves, Molino (N1) and Caballo Moro (N2) and in the Micos area we sampled one of three closely clustered caves (Río Subterráneo, referred to below as Micos or N3). An overview of the geographical distribution of the sampling area of cave and surface locations is presented in Figure 1and the locality abbreviations are shown in Table 1.

**Table 1:** Sample information and descriptive statistics summary of the sampled populations.

Region	Population	Code	N	n	P	A	Ar	He	Ho	Lat	Long
**EL ABRA**	Pachón	**O1**	45	36.31	0.92	4.81	2.19	0.48	0.47	22.60	99.05
	Yerbaniz	**O2**	12	9.46	0.96	6.00	2.67	0.60	0.60	22.20	98.97
	Japonés	**O3**	10	7.77	0.92	4.12	2.55	0.57	0.55	22.10	98.95
	Arroyo	**O4**	12	9.19	0.88	3.62	2.36	0.53	0.50	22.20	98.97
	Tinaja	**O5**	4	3.56	0.88	2.72	2.5	0.56	0.58	22.08	98.95
	Curva	**O6**	13	10.00	0.88	3.75	2.3	0.43	0.49	21.98	98.93
	Toro	**O7**	3	2.69	0.77	2.23	2.98	0.60	0.55	21.85	98.93
	Chica	**O8**	119	104.08	1.00	9.27	2.74	0.64	0.60	21.85	98.93
	Mean statistics of the group		27	22.88	0.90	4.56	2.54	0.55	0.54		
**GUATEMALA**	Molino	**N1**	22	19.31	0.85	3.04	1.89	0.40	0.39	23.06	99.16
	Caballo Moro	**N2**	26	22.69	1.00	5.73	2.6	0.58	0.53	22.92	99.20
	Mean statistics of the group		24	21	0.92	4.38	2.25	0.49	0.46		
**MICOS**	Subterráneo	**N3**	72	58.88	1.00	10.96	2.98	0.66	0.57	22.10	99.18
**SURFACE**	Río Frío	**S1**	10	7.08	1.00	6.64	3.71	0.72	0.73	22.99	99.15
**STREAMS**	Arroyo Sarco	**S1**	32	27.42	1.00	11.62	3.57	0.82	0.82	22.02	99.32
	Chamal	**S2**	13	7.38	0.96	6.62	3.35	0.83	0.75	22.84	99.20
	Río Meco	**S2**	27	19.27	1.00	10.00	3.67	0.81	0.74	22.82	99.31
	Río Tantáon	**S3**	28	21.50	1.00	11.96	3.52	0.85	0.74	22.37	98.90
	Río Florído	**S3**	15	9.60	1.00	7.92	3.53	0.80	0.73	21.98	98.77
	RióTampaón	**S3**	26	17.23	1.00	10.15	3.74	0.84	0.77	21.85	98.94
	Nacimiento del Río Santa Clara	**S3**	24	19.62	1.00	11.04	3.7	0.85	0.82	22.50	98.9
	San Rafael Los Castros	**S3**	25	19.62	1.00	11.19	3.65	0.84	0.76	22.75	99.02
	Rio Subterráneo Valley	**S4**	30	22.88	1.00	11.08	3.83	0.83	0.76	22.13	99.17
	Mean statistics of the group		23	17.16	1.00	9.82	3.63	0.82	0.76		

N = sample size per population; n = mean sample size over all loci; P = proportion of polymorphic loci; A = mean number of alleles per locus; Ar = mean number of alleles standardized to the smallest sample. H_e _= unbiased expected heterozygosity standardized according to the (2N/2N-1)*H_e_) formula; H_o _= observed heterozygosity, Lat is the latitude (N) and Long is the longitude (W).

DNA extraction and genotyping were done according to Protas *et al. *(2006). All samples were profiled at 26 microsatellite markers with primers previously developed for QTL studies [10]. The forward primer of each pair was labeled at the 5' end with a fluorescent dye (HEX or FAM) and microsatellite amplification products were visualized on an ABI 3730 automated DNA sequencer. Microsatellite markers were optimized for the allelic range and multiplexed. Allele sizes were scored using v3.7 (ABI). We used unlinked markers selected from independent linkage groups, or markers so distant as to assort independently if within the same linkage group [9]. In addition, all 26 of the markers (Additional File 1) were chosen such that they were outside the previously identified QTL regions [9,10,12].

The database was optimized for analysis using the coalescent-based program Migrate-N 3.2.6. [18-20]. The accuracy of estimates based on the coalescent approach depends more on number of independent loci than on sample size [21]. Thus, our choice of 26 independently assorting loci, biased towards neutrality, maximized the amount of information per unit effort we could extract from the analyses. Furthermore, parameter estimates from Migrate-N 3.2.6 are uninfluenced by missing data. Therefore, we examined all GENEMAPPER calls by hand to verify their validity. Amplifications that were too weak to resolve the peaks or had extra peaks were reamplified and rerun to resolve the problem. Any remaining unresolved were treated as missing data. The overall data set has approximately 20% missing data, but gives unbiased estimates of parameters based on Migrate-N 3.2.6. In order to check for the existence of null alleles, and to evaluate their impact on the estimation of genetic differentiation we used the program MICRO-CHECKER v2.23 [22].

### Genetic diversity

We calculated observed (H_o_) and unbiased expected (H_e_) heterozygosities [23], number of alleles, and the number of alleles standardized for the smallest sample size for single populations and for the geographic groups. These descriptive statistics were performed in Genepop v 4.0 [24] and Microsatellite Analyzer (MSA) [25]. Deviations from HWE were estimated using both the exact test and the F_IS _statistic estimations, using Markov chain Monte Carlo (MCMC) runs for 1000 batches, each of 2000 iterations, with the first 500 iterations discarded before sampling [26]. Whenever multiple testing was performed, probability values were corrected using standard Bonferroni corrections [27].

### Population structure analysis and differentiation

The program STRUCTURE 2.3.3 [28] was used to infer historical lineages through clustering of similar genotypes. The admixture model of STRUCTURE and the option of correlated allele frequencies between populations were used. The correct number of clusters (K) was determined by testing K values from 1 to 12 and performing 10 repeats for each K. The burn-in period consisted of 1 × 10^6 ^iterations followed by 1 × 10^5 ^MCMC repeats. Finally, estimated log probabilities of data Pr (X | K) for each value of K were evaluated by calculating ΔK, the rate of change in the log probability of data between successive K values [29]. We also estimated population structure independently with STRUCTURAMA [30] in order to test the STRUCTURE inferences.

While these clustering methods can be quite powerful, particularly when there is a high divergence between populations [31], they often make explicit assumptions of demographic history and sometimes are difficult to interpret without background biological information. Thus, we complemented the Bayesian analysis using other methods to more directly estimate relationships among populations. The proportions of shared alleles between populations were calculated in the R package adegenet 1.2-2 using the propShared function [32] where the average proportions of shared alleles among and within populations are computed over all possible combinations of individuals sampled. The distance matrix based on the proportion of shared alleles was then transformed into a matrix of Euclidean distances using the quasieuclid function.

Private allele estimates and allele richness were calculated, grouping the independent geographical regions obtained by clustering methods. In order to estimate rarified allelic richness and private rarified allelic richness, the rarified method in HP-RARE [33] was used to control for the correlation between observed allelic diversity and sample size [34]. The alleles were rarified to a sample size of 40, the smallest sample size of our population groups.

In order to estimate the variance between the groups of populations, pooled sample structuring was estimated using analysis of molecular variance (AMOVA) [35] and 20,000 permutations implemented in Arlequin v 3.5.1.2 [36]. Missing data as observed in our study could influence AMOVA results. Thus, locus-by-locus AMOVA was used to adjust the sample sizes for each locus and the point estimators of variance components to estimate F-statistics more accurately [35]. Influences of long-term separation and genetic drift were measured by comparative methods of allelic frequency tests for all population combinations using F_ST _pairwise estimates [37] as implemented in MICROSATELLITE ANALYSER (MSA) [25].

### Migration patterns between populations

The coalescence-based program MIGRATE-N 3.2.6. [18-20] was used to test for and estimate gene flow between populations. Three migration models were evaluated: (1) a full model with two population sizes and two migration rates (in and out of the caves); (2) a model with two population sizes and one migration rate (gene flow into the caves); (3) a model with two population sizes and one migration rate (gene flow out of the caves).

MIGRATE-N 3.2.6 [18-20] also estimated the mutation-scaled effective population size Θ = 4Neμ, where N_e _is the effective population size and μ is the mutation rate per generation per locus, as well as mutation-scaled migration rates M = m/μ, where m is the immigration rate per generation among populations. The model comparison was done using Bayes factors that need the accurate calculation of marginal likelihoods. These likelihoods were calculated using thermodynamic integration in MIGRATE-N 3.2.6 [20] (Additional File 2).

### Mantel Test

We tested for isolation by distance among populations and sample locations comparing genetic distance (F_st_/(1-F_st_)) *versus *straight line geographic distance by application of the Mantel test as implemented in GENALEX [38] (999 permutations, significance level p < 0.01).

### Eye size measurements

In order to test whether variation in eye rudiment size was correlated with genotype in the caves with phenotypically mixed populations, we analyzed digital images of individuals from three cave populations (N2 (n = 26), N3 (n = 72) and O8 (n = 119). Photos were taken in the lab using a digital camera with the fish placed on a Cartesian coordinate grid. Measurements were made using ImageJ (NIH). In order to correct for individual size differences, relative eye size was standardized as a proportion of standard body length [39].

## Results

### Genetic Diversity

We calculated descriptive statistics using 26 unlinked microsatellite markers. The number of alleles and proportions of polymorphic loci were generally higher in surface than in cave populations, although there was considerable variability among populations (Table 1). Genetic variability was significantly lower in the cave populations than in the surface (Table 1). Average allelic number (Ar) ranged from 2.25 ± 0.50 in the Guatemala region (N1, N2) and 2.54 ± 0.26 in the El Abra (O1 to O8), to a high of 3.63 ± 0.14 in the surface populations. Surface Ar was significantly greater than Ar in the Guatemala and in the El Abra (t_16 _= 11.6, t_10 _= 8.7, respectively, P < 10^-6 ^for both). The Micos cave population (N3) had an intermediate average number of alleles per locus (2.98); previous studies have shown this population to contain both cave and surface-dwelling phenotypes [7,16]. We also detected monomorphic loci (NYU26, 26C, 218A, 213B), which shared the same alleles among El Abra cave populations (Additional File 3). Unbiased *e*xpected heterozygosities (H_e_) were also higher and significantly different (0.82 ± 0.04) in surface populations than in the El Abra (0.55 ± 0.07; t_16 _= 10.8, P < 10^-6^) or Guatemala (0.49 ± 0.13; t_10 _= 8.7, P < 10^-5^) populations, while the Micos population (N3) exhibited intermediate heterozygosities of 0.66 (Table 1).

### Genotypic frequencies

We tested all the loci used in the study for the presence of non-amplifying alleles (null alleles) as described in MICRO-CHECKER v2.23 (Van Oosterhout et al. 2004). This method is mostly based on significant heterozygote deficits relative to HWE (Van Oosterhout et al. 2004). However biological factors such as Wahlund effect or inbreeding might be easily misconstrued as evidence for null alleles (Chakraborty et al. 1992). Null alleles seem to be present mostly in the Micos and Chica populations (data not shown). If, however, null alleles were the cause of the lack of heterozygotes, we would expect them to be equally represented in all the other populations, as well as locus-specific; this we did not observe. Thus, loci out of HWE owing to heterozygous deficit seem to be caused not by a technical artifact such as null alleles, but rather by population genetic phenomena. Only one locus (213B) was out of HWE in many populations (9 out of 21) (Additional File 3). Thus, data were analyzed both including and excluding this locus. This made no difference, so the locus was retained.

We performed 519 tests (27 values were excluded due to missing or monomorphic data) and detected 71 significant departures from HWE (based on 0.05 level of significance and standard Bonferroni corrections). Most of the significant loci showed heterozygote deficiency characterized by a positive F_IS _value. Heterozygote excess was detected in a few populations, mostly surface, for five loci (214D, 210A, 202D, 104A and 241B) (Additional File 3). Populations previously described in the literature [7,40], as phenotypically mixed, O8 and N3, presented significant deviations from HWE at numerous loci: 13 and 16 out of 25 loci scored, respectively (Additional File 3). Presumably, this reflects population subdivision. The other cave populations exhibited only small numbers of loci out of HWE and these differed from one population to the next (Additional File 3). One locus (213B) was out of HWE in many populations (9 out of 21) (Additional File 3), which may reflect the presence of null alleles at this locus.

### Population structure analysis and differentiation

**Figure 2:**
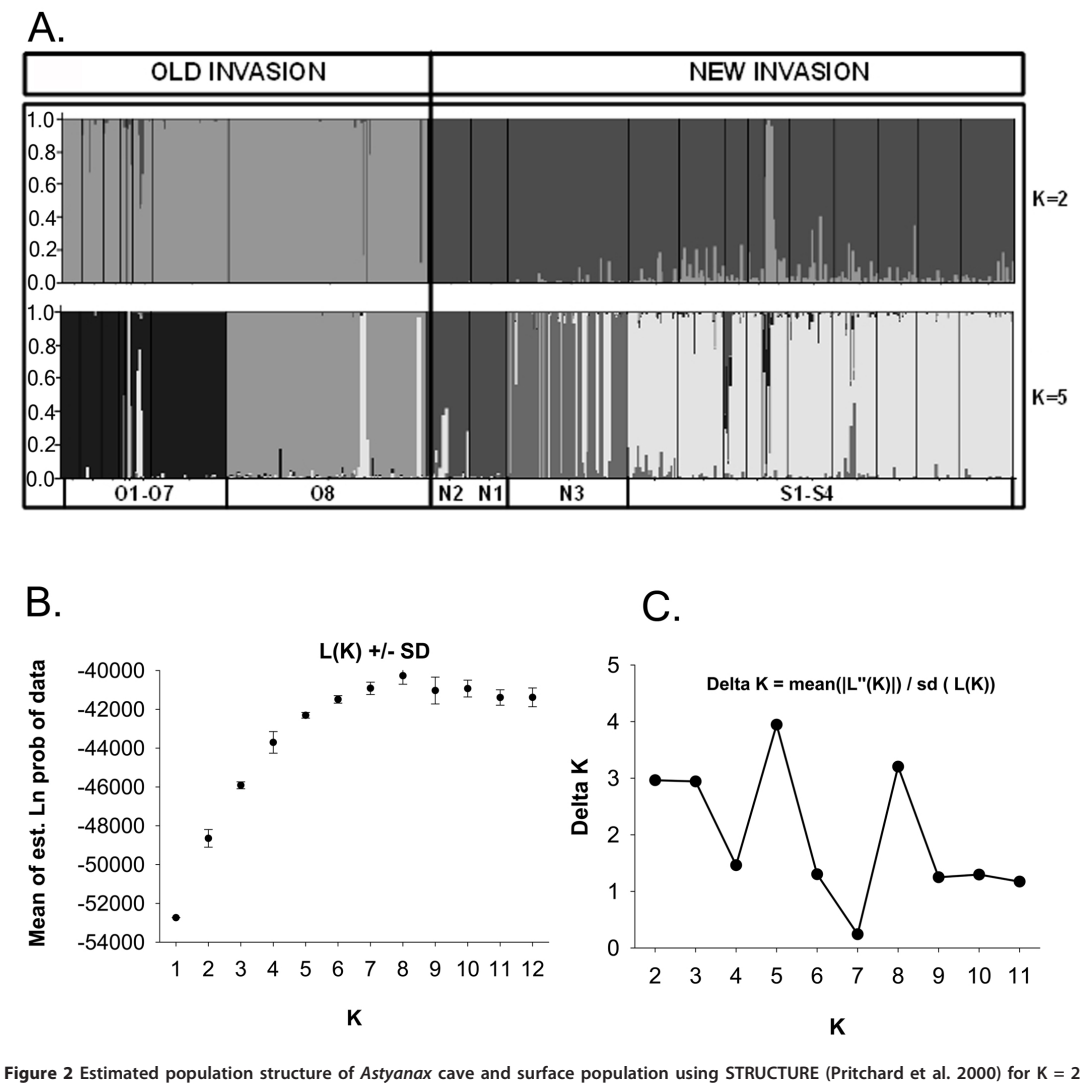
**Estimated population structure of *Astyanax *cave and surface population using STRUCTURE (Pritchard et al. 2000) for K = 2 and K = 5 population groups (Figure. 2)**. Each individual is represented by a thin vertical line, which is partitioned into K segments that represent its estimated population group membership fractions. Black lines separate individuals from geographical site locations (labeled below), which are as following: El Abra: O1 - O7; Chica (O8); Guatemala: N1 - N2; Micos: N3: Surface: S1 - S4. Figure 2B. Mean posterior probabilities of ten runs for each K, K = 1 to K = 12. Figure 2C. K = 5 had the highest ΔK vs. K peak height [29]).

As a starting point to infer the relationships among populations we used the clustering algorithm implemented in the program STRUCTURE [28]. We explored different numbers of populations K to uncover hierarchical population structure (Figure 2A). The clear distinction among the two groups when K = 2 is consistent with the hypothesis that all of the populations we studied originated from two stocks: a "new" stock including present-day surface-forms and the "new" cave populations from the Micos (N3) and Guatemala regions (N1, N2), and an "old" stock including the El Abra cave populations (O1-O8) and their locally extinct progenitors [14-17]. Further structuring represents divergence of O8 from the other El Abra populations at K = 3, the more recent divergence between the surface populations (S1 - S4) and the new cave populations (N1 - N3) at K = 4, and the separate origins of the new cave populations at K = 5. Optimal K [29] estimated the most likely number of populations at K = 5 (Figure 2B, C). We performed a STRUCTURAMA analysis, which estimated the same value of K = 5 (posterior probability of 90%; results not shown).

These five independent population groups are: 1) El Abra caves (O1-O7), 2) El Abra cave mixed population (O8), 3) the new cave populations to the north in the Guatemala region (N1, N2), 4) the new cave mixed population in the southwest Micos region (N3) and 5) the surface populations (Figure 2A and 2C). The STRUCTURE analysis also revealed that four of the cave populations (N3, O8, N2 and O2) contained alleles from surface populations at several loci while the surface populations showed a smaller number of the alleles from the caves.

**Figure 3:**
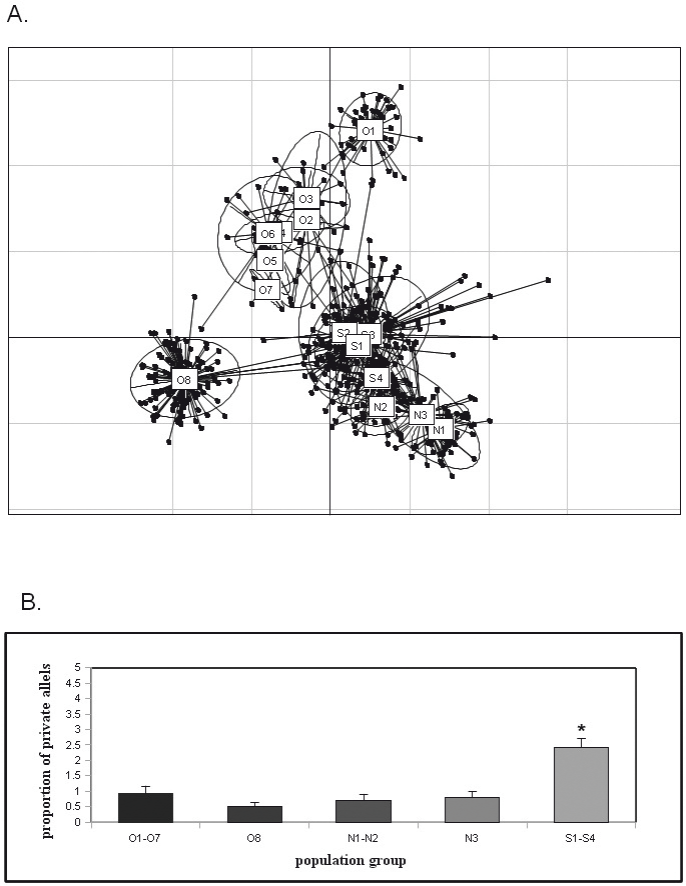
**Genetic variability in *Astyanax mexicanus *using 26 microsatellite loci**. Figure 3A. Proportion of shared alleles (samples of likely common ancestry determined by shared alleles) between the studied populations shown as Euclidian distances, 95% confidence ellipses represent each population. Figure 3B. Private allelic richness averaged over geographically grouped populations. Populations are coded as follows: El Abra caves (O1 - O7); Guatemala (N1 - N2); Micos (N3), Chica (O8); Surface (S1 - S4). All bar plots represent mean ± SEM. Asterisk denotes that the surface group was significantly different than each of the other groupings at the P < 0.0001, as tested by Student's t.

We further tested the genetic distances among populations using the metric of shared alleles. Figure 3A illustrates that the entire El Abra cluster is the furthest away from the cluster of the "new" caves (N1, N2 and N3). Genetically, one El Abra cave population (O8) was equidistant from the "old" and "new" lineages, while the Micos (N3) cave population shared the most alleles with surface populations (Figure 3A).

Private allele estimates were calculated based on groupings of populations united by geographical proximity, which also corresponded well to the groupings revealed both by STRUCTURE and the shared allele distance analysis. The private allele content is significantly higher in surface populations than in cave populations (Figure 3B) (Kruskal-Wallis H test, df = 4, N = 125, H = 32.8, P < 0.00001 for overall significance, post-hoc comparisons revealed no significant differences among cave groups, but all cave groups differed from surface at P < 0.001). The shared alleles and private allele proportions between surface and cave populations (Figure 3A and 3B) suggests that the allelic contents of cave populations are largely subsets of alleles of the surface stock. Thus the observed variation in the caves is mostly the result of standing genetic variation from the ancestral surface stock as well as possible gene flow between the populations (Figure 3B). However, this result needs to be put into perspective: since the ancestors of the 'old cave fish' in the Sierra El Abra are locally extinct, one cannot compare their allelic composition with that found in today's El Abra cave populations.

In order to determine genetic structuring in the analyzed samples, we performed hierarchical AMOVA analysis (Table 2). First, we narrowed down the population structuring by grouping populations based on their origins, "old" *vs*."new" [14-17]. Comparison of the El Abra populations (O1-O8) *vs*. the Guatemala and Micos caves (N1 - N3) pooled with surface populations (S1 - S4) was significant (P < 0.0001) and explained 4.52% of the variance among groups. This supports the hypothesis emerging from the STRUCTURE analysis that two different stocks of surface fish were ancestral to the present day cave populations. We tested by AMOVA all inferences of structuring that emerged from the shared allele distance and STRUCTURE analyses. The largest proportion of variance in all of the groups was within individuals (Table 2). We found no significant structure of the surface populations comparing the regions S1 through S4 (data not shown). Importantly, the AMOVA analyses supported significant structure among six metapopulations: 1) the El Abra cave populations (O2 -O7), 2) Guatemala populations (N1 and N2), 3) Micos population (N3), 4) Chica population (O8), 5) Pachon population (O1) and 6) surface populations.

**Table 2:** Analyses of molecular variance (AMOVA) in cave and surface populations for 26 microsatellite loci

Structure tested	SS	VC	%VAR	Fstat	P
**TWO GROUPS: O1-O8 *vs*. S1-S4 + N1-N3**					
Among groups	372.71	0.43	4.52	0.07	**< 0.000001**
Among populations within groups	1288.50	1.51	15.76	0.16	**< 0.000001**
Among individuals within populations	3743.17	0.58	6.02	0.04	**< 0.000001**
Within individuals	3373.50	7.06	73.70	0.26	**< 0.000001**
**THREE GROUPS: O1-O8 *vs*. N1-N3 *vs*. S1-S4**					
Among groups	560.21	0.45	4.73	0.05	**< 0.000001**
Among populations within groups	1100.99	1.41	1.41	0.16	**< 0.000001**
Among individuals within populations	3743.17	0.58	6.08	0.08	**< 0.000001**
Within individuals	3373.50	7.06	74.31	0.26	**< 0.000001**
**FOUR GROUPS: O1-O7 *vs*. N1-N3 *vs*. S1-S4 *vs*. O8**					
Among groups	575.99	0.67	6.95	0.06	**< 0.000001**
Among populations within groups	1085.21	1.32	13.75	0.14	**< 0.000001**
Among individuals within populations	3743.17	0.58	5.99	0.07	**< 0.000001**
Within individuals	3373.50	7.06	73.30	0.26	**< 0.000001**
**FIVE GROUPS: O1-O7 *vs*. N1-N2 *vs*. S1-S4 *vs*. N3 *vs*. O8**					
Among groups	1034.04	0.86	9.05	0.13	**< 0.000001**
Among populations within groups	539.32	1.20	12.64	0.09	**< 0.000001**
Among individuals within populations	3191.16	0.59	6.19	0.07	**< 0.000001**
Within individuals	2852.00	6.83	72.11	0.27	**< 0.000001**
**FIVE GROUPS: O2-O8 *vs*. N1-N2 *vs*. S1-S4 *vs*. N3 *vs*. O8 *vs*. O1**					
Among groups	1198.68	1.18	12.42	0.12	**< 0.000001**
Among populations within groups	374.68	0.89	9.39	0.1	**< 0.000001**
Among individuals within populations	3191.16	0.59	6.18	0.07	**< 0.000001**
Within individuals	2852.00	6.83	72.00	0.28	**< 0.000001**

SS - Sum of squares; VC - Variance components; % VAR - Percentage of variation; Fstat = F-statistics; P = P values.

Pairwise F_ST _comparisons of the geographically defined populations typically revealed higher divergences among cave populations, even within a geographical cluster, than between cave and surface populations (Table 3). F_ST _comparisons revealed less divergence among populations of the two Guatemala caves (N1, N2) and Micos cave (N3) (F_ST _range from 0.23 to 0.36) than was seen in comparisons among caves of the El Abra cluster (F_ST _range from 0.20 to 0.51). This low genetic divergence is notable because the Sierra de Guatemala and Micos caves are more than 100 kilometers (km) apart. Consistent with the AMOVA analyses, F_ST _values among surface populations were generally low (the highest F_ST _= 0.09), suggesting that many of these populations from multiple and distant geographical regions essentially have high levels of allelic exchange. On the basis of F_ST _values, general divergence between cave and surface pairs seems to be related to the level of the physical isolation of the particular caves from the surface water. Four cave populations (O1, O6, N1, and N2) show the highest F_ST _values against the surface populations (Table 3). The first three of these populations are perched and thus isolated from the underlying aquifer, while the fourth is in an area with no permanent surface streams (Additional File 4). Results of a Mantel test for isolation by distance among populations O1 to O8 were positive, showing increasingly greater genetic isolation with increasing geographic distance (Additional File 5).

**Table 3:** Multilocus pairwise F_ST _estimates from 26 microsatellite loci in *Astyanax mexicanus*.

	EL ABRA								GUATEMALA		MICOS	SURFACE STREAMS								
	**O4**	**O6**	**O3**	**O5**	**O7**	**O2**	**O1**	**O8**	**N2**	**N1**	**N3**	**S3**	**S2**	**S1**	**S3**	**S2**	**S3**	**S3**	**S3**	**S1**

**O6**	**0.06**																			
**O3**	**0.19**	**0.25**																		
**O5**	0.06	0.15	0.21																	
**O7**	0.08	0.12	0.15	0.08																
**O2**	**0.14**	**0.23**	0.05	0.16	0.11															
**O1**	**0.28**	**0.34**	**0.31**	**0.30**	**0.34**	**0.28**														
**O8**	**0.22**	**0.23**	**0.26**	**0.18**	**0.16**	**0.23**	**0.33**													
**N2**	**0.35**	**0.36**	**0.33**	**0.33**	**0.31**	**0.33**	**0.41**	**0.29**												
**N1**	**0.47**	**0.51**	**0.44**	**0.47**	0.46	**0.46**	**0.48**	**0.37**	**0.36**											
**N3**	**0.24**	**0.26**	**0.26**	**0.23**	**0.20**	**0.24**	**0.31**	**0.23**	**0.23**	**0.27**										
**S3**	**0.16**	**0.19**	**0.15**	**0.13**	**0.08**	**0.14**	**0.23**	**0.16**	**0.19**	**0.26**	**0.11**									
**S2**	**0.21**	**0.26**	**0.19**	0.20	0.09	**0.18**	**0.30**	**0.18**	**0.20**	**0.33**	**0.12**	0.00								
**S1**	**0.23**	**0.30**	**0.25**	0.20	0.13	**0.22**	**0.31**	**0.20**	**0.26**	**0.39**	**0.19**	**0.05**	**0.07**							
**S3**	**0.18**	**0.22**	**0.18**	0.17	0.10	**0.18**	**0.26**	**0.15**	**0.22**	**0.34**	**0.11**	0.02	0.03	**0.09**						
**S2**	**0.19**	**0.22**	**0.17**	**0.17**	**0.10**	**0.17**	**0.25**	**0.19**	**0.20**	**0.28**	**0.13**	**0.02**	0.01	**0.08**	**0.04**					
**S3**	**0.17**	**0.21**	**0.17**	**0.15**	**0.10**	**0.15**	**0.24**	**0.15**	**0.19**	**0.25**	**0.08**	0.01	0.02	**0.06**	0.02	**0.04**				
**S3**	**0.17**	**0.21**	**0.16**	**0.14**	0.09	**0.14**	**0.25**	**0.14**	**0.19**	**0.28**	**0.11**	0.00	0.01	**0.05**	0.02	**0.03**	0.01			
**S3**	**0.18**	**0.22**	**0.18**	**0.15**	0.09	**0.16**	**0.26**	**0.16**	**0.19**	**0.28**	**0.13**	0.00	0.02	**0.03**	**0.03**	**0.04**	0.01	0.00		
**S1**	**0.18**	**0.22**	**0.18**	**0.16**	**0.11**	**0.16**	**0.25**	**0.16**	**0.20**	**0.28**	**0.13**	**0.02**	**0.03**	**0.05**	**0.03**	**0.05**	**0.02**	0.01	0.01	
**S4**	**0.19**	**0.22**	**0.18**	**0.16**	0.11	**0.17**	**0.26**	**0.17**	**0.19**	**0.26**	**0.07**	**0.03**	**0.04**	**0.09**	**0.04**	**0.04**	**0.02**	**0.03**	**0.04**	**0.06**

Boldfaced values are significant after Bonferroni correction.

As is the case with O1, all seven F_ST _values between O8 and the other old cave populations are significant. In fact, F_ST _analyses reveal that the O8 population is significantly diverged from every other population of cave or surface fish we surveyed (Table 3). The average F_ST _value between O8 and the seven other cave populations of the El Abra group (O1 to O7) was 0.230 ± 0.021 (SEM), which was significantly higher than the average F_ST _values between O8 and the ten surface populations (average F_ST _= 0.166 ± 0.006; t_11 _= 5.75, p < 0.0005).

### Effective population size and migration rates in *Astyanax mexicanus*

**Figure 4:**
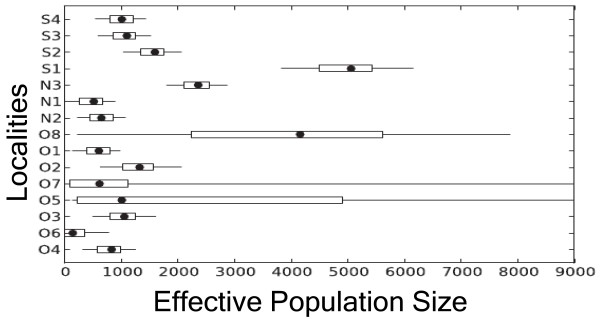
**Estimates of effective population size (N_e_) based on Bayesian inferences of migration rates and population sizes among *Astyanax mexicanus *population**. The central box of the plots represents the values from the lower to upper quartile (25 to 75 percentile). The middle dot represents the median posterior values over all loci. The horizontal line extends from the 2.5% percentile to the 97.5% percentile. The x-axis represents N_e_. Populations are coded as follows: El Abra caves (O1 - O7); Guatemala (N1 - N2); Micos (N3), Chica (O8); Surface (S1 - S4).

**Figure 5:**
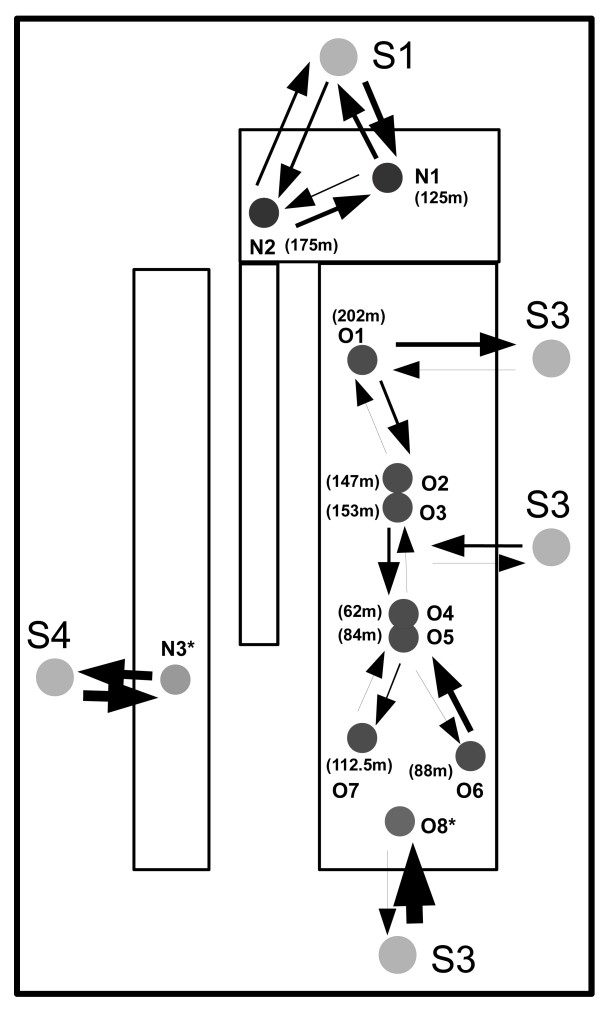
**Summary of the estimates of gene flowbased on Bayesian inferences of migration rates and population sizes using MIGRATE-N 3.2.6 among *Astyanaxs mexicanus *population clusters within each geographical region**. The arrows represent directions of migration and the thicknesses are proportional to the M (the ratio of immigration rate and mutation rate). Populations are coded as follows: El Abra caves (O1 - O7); Guatemala (N1 - N2); Micos (N3), Chica (O8); Surface (S1 - S4). Asterisk denotes mixed populations.

Estimations of effective population sizes (N_e_) and migration rates among populations were performed with MIGRATE-N 3.2.6, using Bayesian inference and the Brownian motion mutation model. The model allows for mutation rates differing among loci by using the number of alleles per locus to estimate locus specific relative mutation rate modifiers. All the estimates of the mutation-scaled effective population size Θ were scaled using a microsatellite mutation rate of 5.56 × 10^-4 ^per locus per generation [41,42] to calculate the average effective population sizes (N_e_). Effective population sizes varied among different surface clusters (N_e _from ~1011 to ~5058) but were generally greater than in cave populations (Figure 4). Estimates of N_e _in most cave populations ranged from 831 (O6) to 1326 (O2) (Figure 4). However, the cave populations from which previous studies reported mixed populations were again an exception, with effective population sizes of 4159 in O8, 1326 in O2, and 2360 in N3. We used the MIGRATE-N 3.2.6 models [20] to test for gene flow among individual cave and surface populations, limiting our inquiry to nearby populations or adjacent cave clusters. The summary of all the models is in Figure 5 (Additional File 6 for the details). Our estimations of migration rates and effective population sizes supported the hypothesis that the genetic diversities of *A. mexicanus *cave populations are functions of introgression from surface populations, as well their effective sizes.

Migration rates between individual populations varied by several orders of magnitude and the rates between cave and surface populations exceed those between caves. This is in accord with calculated F_ST _values. Four different patterns of migration were observed: among surface populations, among cave populations, from cave to surface, from surface to cave. Migration rates among the four groups of surface populations defined earlier (S1 - S4) were the highest we observed and were mostly symmetrical (Additional File 6). Migration rates between cave and surface populations were largely asymmetrical, with migration from the surface into caves typically greater than in the reverse direction. Micos (N3) cave and its nearby surface population was the only case in which migration rates in both directions were nearly equal, a result consistent with the STRUCTURE results. Migration rates among the cave populations were very low, except for caves in the El Abra or Guatemala clusters that are in close geographic proximity (O2 - O3; O4; O5 - O6; N1 - N2). Also, N1 - N3 seem to have more exchange of migrants with surface than with populations of the old cave cluster (Figure 5, Additional File 6). This suggests that proximate caves can exchange alleles through migration, although not nearly to the same extent as the surface populations exchange alleles.

Considering only the O1 - O8, we see that migration rates decrease with increasing geographical distances among populations (Figure 5, Additional File 4). This observation supports the hypothesis of underground connections between nearby populations. Thus, O1 as the most geographically distant cave has the smallest influx from other cave populations of El Abra cluster, while O2 - O3 and O4 - O5 show high gene flow in both directions (Figure 5, Additional File 6).

In some cases the estimates of gene flow between two caves or cave clusters appear asymmetric. Considering both the Sierra de El Abra and the Guatemala, these asymmetries seemed related to relative altitudes. Figure 5 shows the altitudes above sea level of the fish pools in the various caves; N2 (175 m) sent more migrants to N1 (125 m) than vice versa. The same is true for O1 (202 m) to O2/O3 (147/153 m), O2/O3 to O4/O5 (62/84 m), and O7 (88 m) to O4/O5. Thus, we suggest it is easier for migrants to move downstream than upstream.

It must be noted that many of the estimates of migration rates are associated with large error terms (Additional File 6) and are not precise. Nevertheless, the overall trends discussed above seem clear.

### Relationship between eye phenotype and individual admixture proportions

**Figure 6:**
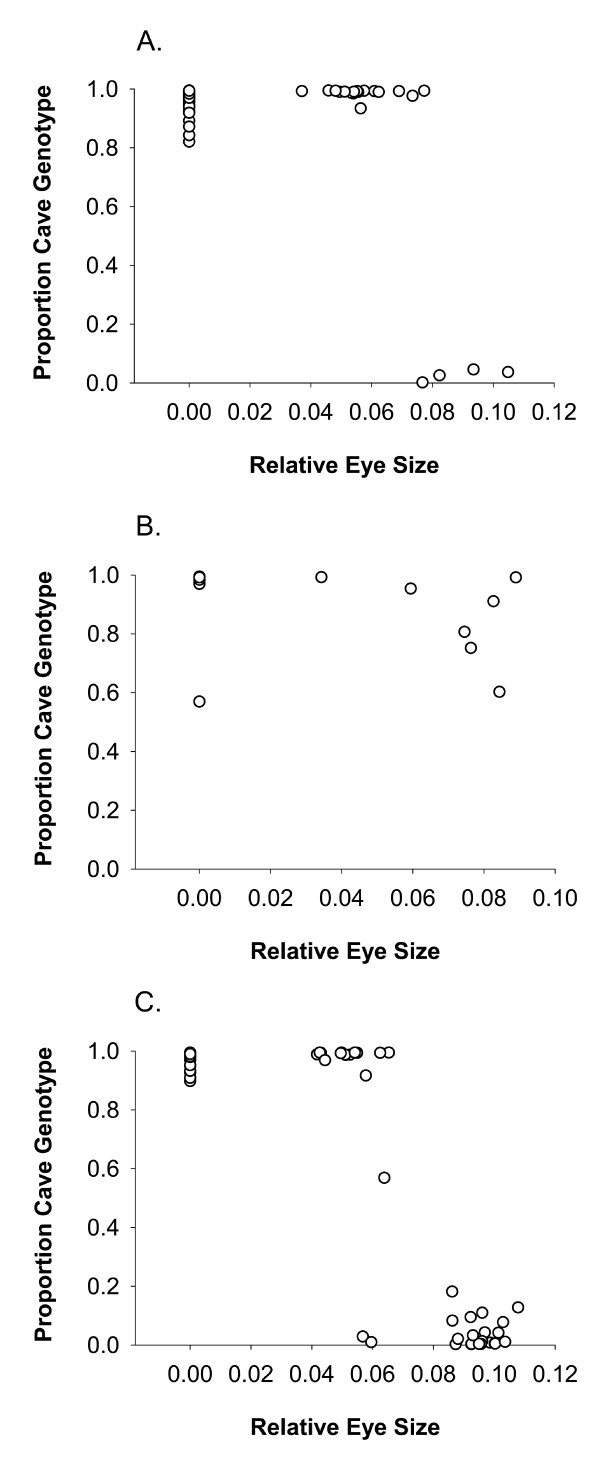
**Correlations between genotype and phenotype in three mixed cavefish populations**. Each point represents an individual fish. Phenotype is represented by relative eye size and genotype as the admixture proportions from the STRUCTURE analysis. A represents O8, B represents N2, C represents N3.

In order to understand the integration of the surface individuals into the cave in our populations we compared phenotype and genotype for individuals collected from the three caves with mixed populations (O8, N2 and N3). The phenotype we used was relative eye size and the genotypic designations for each of the 26 loci were obtained from the STRUCTURE analyses (Figure 6). Hybrids between cave and surface formes are intermediate in phenotype between the two [6]. Our results largely represent sorting of the phenotype and genotype into the two main categories, surface and cave. In addition, however, we also observe that there are individuals that are in intermediate states in both genotype and phenotype, the expected state for hybrids between surface and cave [9,39,43].

## Discussion

### The Origins of the Cave Populations

Our data clearly show that the populations of cave adapted *Astyanax *in NE Mexico are derived from two separate stocks. Previous studies using microsatellites and mtDNA markers had also concluded that the cave populations were derived from at least two surface stocks [14-17]. Our results clarify the affinities of the Pachón (O1) and Chica (O8) cave populations. Pachón was previously placed with the new stock based on mtDNA data, but our extensive nuclear DNA data set clearly places it with the old stock. Conclusions based on mtDNA may be misleading because the presence of surface fish in cave populations allows for the possibility of the introgression of surface mitochondria [16]. The affinities of the Chica population are discussed below. Finally, the present study covers the full geographic range of the cave populations and reveals no evidence that the cave populations are derived from more than two clades.

Although derived from only two separate stocks, there are clearly more than two subterranean invasions that established the extant cave populations. All of our structuring analyses support divergence among five groups and are in accord with the hypothesis that the cave populations N1 to N3 were established much later than the populations O1 to O8. Similarities in the microsatellite allele frequencies in the "new" cave populations (Molino, Caballo Moro, and Micos, N1 to N3, in order) and surface populations also confirm that these populations have recently diverged (Additional File 1). With the exceptions of Pachón (O1) and Chica (O8), the shared allele analysis shows that the El Abra populations cluster tightly. In the case of Pachón (O1) the divergence is minor and it is much closer to the old (El Abra) cluster than to the new cave cluster. In contrast, the Chica population (O8) is not obviously aligned with either cluster in the shared allele analysis.

The origin of the Chica population (O8) has been a long standing question in the *Astyanax *literature [7]. Our data strongly suggest that the Chica cave originated from old stock. This interpretation contrasts with a previous one based on mtDNA and a small number of microsatellite loci which suggested that it is phylogenetically young and originated from new stock [16,44]. If Chica were phylogenetically young, however, the STRUCTURE analysis should cluster it with the surface populations, a result not observed. Furthermore, we should see lower F_ST _values between Chica and the new cave populations (N1 through N3) than between Chica and the other El Abra populations (O1 through O7), but the opposite is the case (Chica vs. new: average F_ST _= 0.297 ± 0.041 SEM; Chica vs. other El Abra: average F_ST _= 0.230 ± 0.021) (Table 3). Considering the F_ST _values, the STRUCTURE analysis, and the shared allele distance analysis (Table 3 Figure 2and Figure 3A), all of which show Chica to be considerably differentiated from the rest of the El Abra populations, we suggest that it was derived from an independent invasion of old stock. Because of its southernmost location, it may well be the earliest established of the cave populations.

### Geology and Geography

Knowledge of geology and geography, as well as genetics, is needed to understand the pattern of independent invasions of the underground that established the extant populations. A clear pathway through surface waters from the southernmost end of the El Abra all the way to the area of Pachón cave existed in the past but at present a surface divide separates the ends of the valley [7] (Additional File 4). Pachón cave (O1) at the northern end of the El Abra is 46 km north of Yerbaniz cave (O2). While there is at least one other known cave between the two that might have served as a stepping stone, it seems likely that the underground invasion that established the Pachón cave population was independent of those that established the more southern populations. This argument is based on the expectation that travel from one region to another is much faster through surface streams than through subterranean passages because open waters contain abundant food and provide direct passage, while subterranean routes have low food reserves and their passages may be maze-like. Surface fish can move into caves relatively easily and quickly. We constantly see surface *Astyanax *and other surface species, including *Tilapia*, in certain caves, such as Yerbaniz (O2), Chica (O8) and Micos (N3). The significance of Tilapia's presence is that it was introduced into Mexican waters and only became common in the late 1980's [45]. Therefore, its presence in caves shows how quickly underground populations may be seeded from the surface. Thus, for the most distal populations of a migratory wave, it is far likelier that surface migrants will have reached and colonized a cave long before the arrival of underground migrants from the same source. All seven F_ST _values between Pachón and the other El Abra populations are significant (Table 3), which reflects the current isolation of the cave and, perhaps, a past independent origin.

Considering the new cave populations, the distance between the Micos (N3) cave and the closest of the Guatemala caves, Caballo Moro (N2), is over 90 km and there is one ridge and two open valleys between them. No documented underground route currently exists between the two regions. Thus, the Micos and Guatemala cave clusters likely represent separate invasions.

In summary, we suggest a model with a minimum of five independent origins of cave adapted *Astyanax *in NE Mexico. We envision that the area was originally colonized by surface Astyanax of the old stock which independently established cave populations in the south of the El Abra (O8), in central El Abra (O2 - O7), and in its north (O1). Subsequent to this, the surface stock went extinct locally and was eventually replace by surface fish of the new stock. These gave rise to cave populations in the two geographically distinct regions of the Gautemala (N1 and N2) and Micos (N3) (Additional File 7).

### Allelic diversity, migration and gene flow

Allelic diversities were generally lower in cave populations than in surface populations (Table 1), an observation in accord with previous studies on this species and other fishes [14,16,46-48]. Lower genetic diversity in cave populations than in related surface populations probably reflects smaller effective population sizes because of food and space limitations, but may also reflect possible bottleneck events due to periodic droughts and other environmental fluctuations [7]. It should be noted, however, that the relatively large effective population sizes in Micos (N3) and Chica (O8) were probably overestimated by MIGRATE-N 3.2.6 because they are admixed with the surface populations.

Many of the El Abra caves regularly receive migrants from the surface [7,48], and Chica (O8) is the best known of these [40]. Chica is unusual among *Astyanax *cave populations in receiving a high energy input deriving primarily from two bat roosts located directly above the largest of the fish pools. Breder noted, and we still observe today, that the frequency of surface fish in the pools increases as one goes deeper into the cave, and is highest in Pool 4, at the level of the aquifer and located about one km from the Río Tampaón [40]. All who have studied this cave have surmised that surface fish get into the cave from the river through the aquifer and are able to survive and breed there because of the high energy input from the bat roosts and from debris washed into the cave during the rainy season [7,40]. Thus, Chica draws its occupants from two different source populations that are well differentiated from each other. This admixture results in significant heterozygote deficiencies at numerous loci. That these departures from HWE are due to Wahlund effect is evident from genotype-phenotype correlations observed in our study (Figure 6).

Our collections from the Micos cave (N3) also contained both cave and surface forms and, as in Chica (O8), we observed departures from HWE due to Wahlund effects. In contrast to the situation in Chica, food is not abundant in this cave, thus the surface fish are prone to starvation, leading in most cases to reduced fitness and inefficient mating [7]. Nevertheless, some surface fish washed into this cave may hybridize with the cave population, as revealed by genotype-phenotype correlations (Figure 6). The Caballo Moro (N2) population exhibits a full range of eye sizes and pigmentation, from typical cave to typical surface morphs (Figure 6). This population is in a karst window, a habitat within a cave exposed to light because of passage collapse; the presence of light facilitates the continued survival of surface and hybrid phenotypes [7,49].

The MIGRATE-N analysis also detected relatively high rates of gene flow from the Pachón cave population (O1) to their nearby surface populations, supporting an earlier suggestion of a route for alleles from cave to surface [48] (Figure 5, Additional File 6). Estimation of migration rates and effective population sizes supported the hypothesis that the genetic diversity of *A. mexicanus *cave populations is correlated with the influx of alleles from surface populations, as well as by their effective population sizes [48]. The relatively high rates of migration between cave and surface populations here may not be a rule for cavefish. For example, migration between cave and surface populations of *Poecilia sulphuraria *is relatively low, even though there are few physical barriers to movement [5]. In the case of *Poecilia*, the barrier seems to be the extreme environment of the sulphidic caves, which requires physiological adaptation to high levels of H_2_S, a condition to which cave *Astyanax *are not exposed.

The migration rate analysis revealed that surface fish in the region form a metapopulation, with extensive exchange of genetic material among its component populations. Thus, there is high genetic diversity within and little genetic differentiation among surface populations. In strong contrast, cave populations live under dramatically different ecological conditions and often have lower population densities. MIGRATE-N results also show that the effective sizes of surface populations are generally larger than those of cave populations, consistent with earlier studies based on estimates of nucleotide diversity [48] (Figure 4). Mark and recapture estimates of total population sizes from Pachón (O1) and Yerbaniz (O2) caves were similar to our estimates, with averages of 8.5 × 10^3 ^individuals and broad 95% confidence intervals ranging from about 1.5 × 10^3 ^to 17.0 × 10^3 ^[7]. Our estimates of N_e _in cave population varied from 2.8 to 7.3 × 10^3^, with the exception of Curva (O6) and the admixed populations Micos (N3) and Chica (O8). While consistent with the estimates from mark-recapture studies [7], they are around one order of magnitude higher than previously reported estimates from molecular data [48].

We note that the mutation-scaled immigration rate (M) from surface populations into cave populations often exceeds 1.0 (Additional File 6). With mutation-scaled effective population sizes (θ) on the order of 0.5 to 5, mN_e _(θ*M/4) can exceed 1.0, implying that migration from surface to cave populations could significantly affect allelic frequencies at neutral loci [50]. Nevertheless, cavefish in these populations remain troglomorphic in phenotype in the face of this immigration. This implies that these phenotypes are maintained by selection, although we cannot say whether it is natural selection imposed by the cave environment or sexual selection imposed by mate choice biases against the surface fish [5,51]. Selection may generally be sufficiently powerful to allow population differentiation even in situations in which there is high gene flow [52].

Finally, we note that the five independent invasions of the subterranean habitat documented here imply five instances of striking phenotypic convergence. This highlights the importance of a change in ecology as a strong driver of evolutionary change. This is in accord with studies of freshwater adaptation in *Gasterosteus aculeatus *that document widespread convergences or parallelisms related to ecological shifts [53].

## Conclusions

Our study showed that cave populations of *Astyanax mexicanus *generally have significantly lower genetic variability than surface populations, reflecting the generally lower availability of habitat space and food in the caves. Some of the cave populations were exceptional and had higher genetic diversity, which correlated with their receiving relatively high migration from the surface. We documented significant levels of gene flow between surface and cave populations in both directions. That cave populations could maintain a cave specific phenotypic suite of traits in the face of strong migration from the surface implies strong selection for maintenance of cave phenotype. The results also demonstrate that cave populations in the region studied arose at least five times independently and derive from two different ancestral stocks, implying numerous convergences on the cave phenotype driven by the ecological shift from surface to the underground. Thus, the *Astyanax *cavefish model will continue to be a rich source for study of adaptive evolution.

## Authors' contributions

MB designed the study, collected the specimens, performed all the analyses and wrote the manuscript. PB performed the MIGRATE-N analysis and helped with the data interpretation. FJGL and SEB collected the specimens and helped with the manuscript preparation. RB contributed to the design of the study, the choice of populations and collection of specimens, and the writing of the manuscript. All authors read and approved the final manuscript.

## Supplementary Material

Additional file 1 **Summary statistics for 26 microsatellite loci of *Astyanax mexicanus *populations**. Table summarizing the microsatellite data. Mean sample size over all loci (n); Ap is the mean number of alleles per locus, expected (H_e_) and observed (H_o_) heterozygosity, F_IS_. Bold F_IS_values are estimates significantly different from zero after Bonferroni correction. Click here for file

Additional file 2 **MIGRATE-N 3.2.6, runtime conditions and methods**. Summary of methods and conditions. Click here for file

Additional file 3 **Allelic frequencies of 26 microsatllite loci in the studied populations**. Summary of allelic frequencies for all loci and all populations. Click here for file

Additional file 4 **A detailed hydrological map of the El Abra region**. El Abra region map with the indication of surface and subsurface water divide. Points at, or near, base level (orange line) are indicated by solid circles; fish-inhabited pools by solid circles closer to the high water profile (blue dotted line) (adapted from Mitchell et al. 1977). Click here for file

Additional file 5 **Genetic isolation by distance**. Results of the Mantel test for correlation between genetic distance (Fst/(1-Fst)) and geographic distance for populations of the El Abra region (O1 - O8) (R^2 ^= 0.625, P = 0.01). Click here for file

Additional file 6 **Estimates of gene flow based on Bayesian inferences of migration rates and population sizes**. Results of MIGRATE-N 3.2.6 on *Astyanax mexicanus *population clusters within each geographical region. Mutation scaled immigration rate, M, between different population groups. M is the ratio of the immigration rate over the mutation rate. The central box of the plots represents the values from the lower to upper quartile (25 to 75 percentile). The middle dot represents the median posterior values over all loci. The horizontal line extends from the 2.5% percentile to the 97.5% percentile. Populations compared are designated to the left of the boxes. Click here for file

Additional file 7 **Summary of the proposed models and conclusions of the paper**. Proposed model with five independent origins of cave adapted *Astyanax *in NE Mexico as estimated by the data. The first wave of surface fish led to three independent subterranean invasion events establishing the "old" cave populations. The second wave gave rise to two independent invasions establishing "new" cave populations. The arrows signify that the ancestral stock moved into the area from the south but are not meant as specific routes. Click here for file
