# Supplementary material for: Gene flow and population structure in the Mexican blind cavefish complex (Astyanax mexicanus)
Source: BMC Evol Biol. 2012 Jan 23;12:9. doi: 10.1186/1471-2148-12-9 (PMC3282648; doi:10.1186/1471-2148-12-9)
Supplement: Additional file 2 — MIGRATE-N 3.2.6, runtime conditions and methods. Summary of methods and conditions. [file 1471-2148-12-9-S2.DOC]

Most run parameters for the program MIGRATE-N 3.2.6 were left at default values, but adjustments were made on parameters influencing the run-length, heating, and relative mutation rate, and, of course, to specify different migration models.
 The mutation rate among loci was scaled so that the average rate change of the mutation rate was 1.0. This equalizes the effects of the estimates of the individual loci; relative rates changed for different runs because of data differences, but commonly the minimum (0.0369) and maximum rates (2.32) were rare and most datasets had ranges for the rate of mutation rate change of about 0.6 to 1.5.
 Per locus the first 100,000 steps were discarded, then 2.5 million steps were visited using parallel runs of 100 replicates. These resulted in recorded 50,000 samples that were recorded every 50th step. A step comprises of either a parameter change or a genealogy change. A total of 26 loci yielded samples of 65 million steps. To improve searching and also to calculate marginal likelihoods for the model comparison a heating scheme was applied using 4 changes with temperatures 1.00, 1.50, 3.00, and 1000000.00.
 A random genealogy and parameter settings inferred by an FST-based method where used as start condition. The prior distribution for the parameters was uniform with boundaries appropriate for the parameters and data: Theta priors were bounded between 0 and 50.0 and M priors where bounded between 0.0 and 100.0.
