# Supplementary figures and images for: Gene flow and population structure in the Mexican blind cavefish complex (Astyanax mexicanus)
Source: BMC Evol Biol. 2012 Jan 23;12:9. doi: 10.1186/1471-2148-12-9 (PMC3282648; doi:10.1186/1471-2148-12-9)

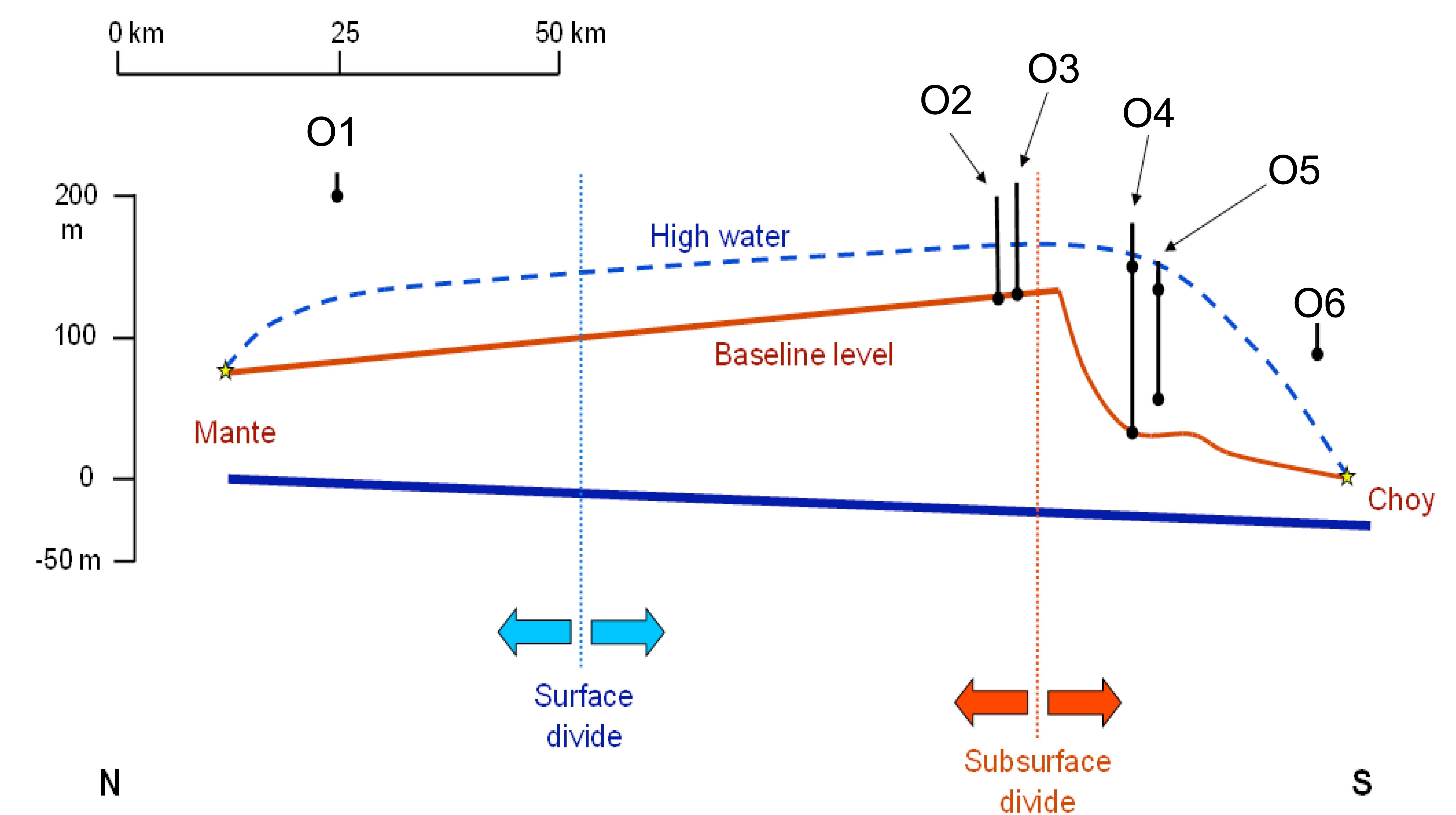

Supplement: Additional file 4 — A detailed hydrological map of the El Abra region. El Abra region map with the indication of surface and subsurface water divide. Points at, or near, base level (orange line) are indicated by solid circles; fish-inhabited pools by solid circles closer to the high water profile (blue dotted line) (adapted from Mitchell et al. 1977). [file 1471-2148-12-9-S4.JPEG]

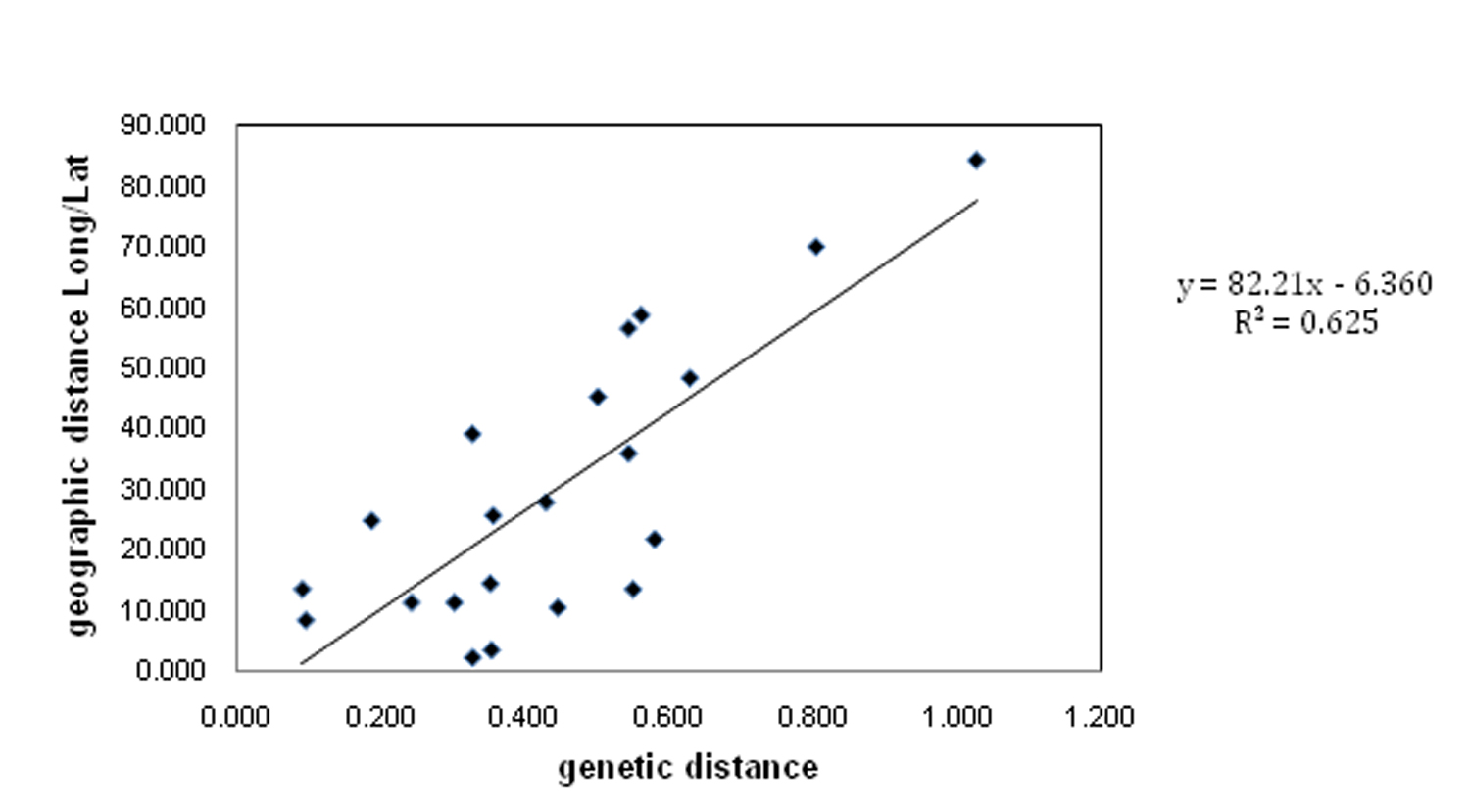

Supplement: Additional file 5 — Genetic isolation by distance. Results of the Mantel test for correlation between genetic distance (Fst/(1-Fst)) and geographic distance for populations of the El Abra region (O1 - O8) (R2 = 0.625, P = 0.01). [file 1471-2148-12-9-S5.JPEG]

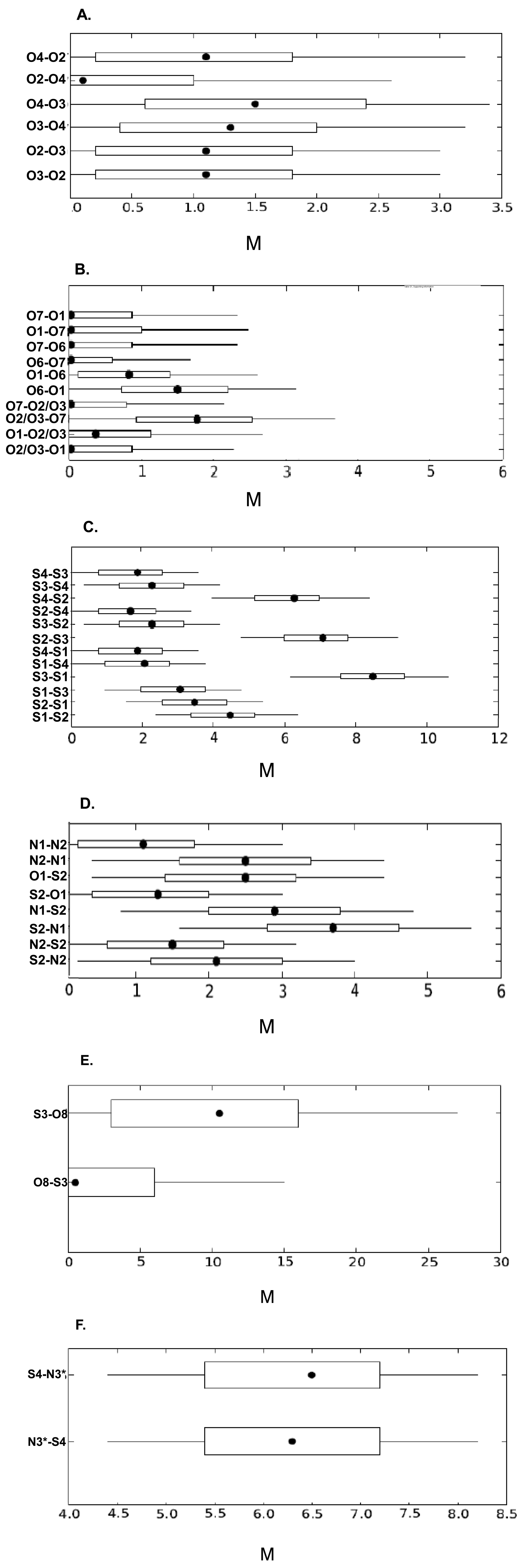

Supplement: Additional file 6 — Estimates of gene flow based on Bayesian inferences of migration rates and population sizes. Results of MIGRATE-N 3.2.6 on Astyanax mexicanus population clusters within each geographical region. Mutation scaled immigration rate, M, between different population groups. M is the ratio of the immigration rate over the mutation rate. The central box of the plots represents the values from the lower to upper quartile (25 to 75 percentile). The middle dot represents the median posterior values over all loci. The horizontal line extends from the 2.5% percentile to the 97.5% percentile. Populations compared are designated to the left of the boxes. [file 1471-2148-12-9-S6.JPEG]

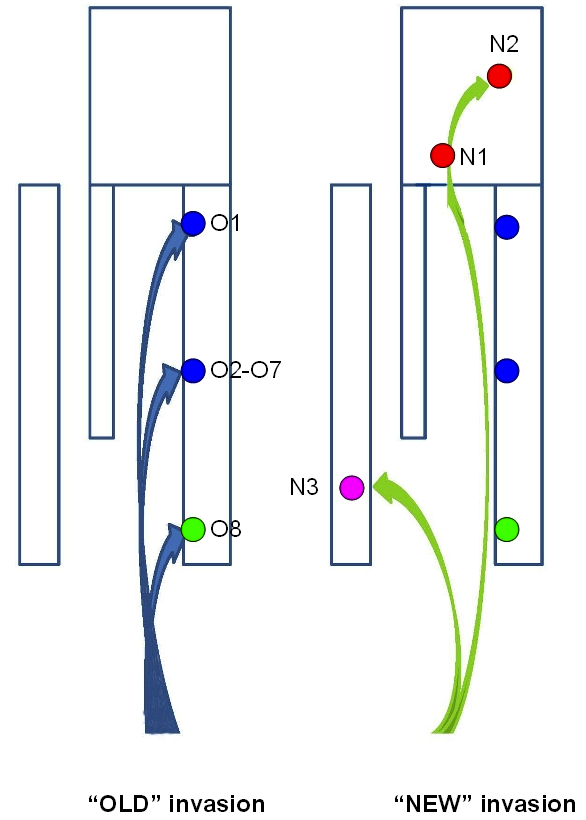

Supplement: Additional file 7 — Summary of the proposed models and conclusions of the paper. Proposed model with five independent origins of cave adapted Astyanax in NE Mexico as estimated by the data. The first wave of surface fish led to three independent subterranean invasion events establishing the "old" cave populations. The second wave gave rise to two independent invasions establishing "new" cave populations. The arrows signify that the ancestral stock moved into the area from the south but are not meant as specific routes. [file 1471-2148-12-9-S7.JPEG]
